# Legislation Run Mad

**Published:** 1897-05

**Authors:** 


"Legislation Run Mad," is what the *Journal of the American Medical Association* (April 10, '97), calls the futile efforts to "regulate the practice" in New York,—or rather—legislation to abate the "hospital and dispensary abuse."

This subject "Hospital and Dispensary Abuse" has been harped upon by the New York medical journals until it is more than threadbare. It has been one long plaintive cry of the much injured doctor,—the "outs,"—whose "practice has been taken away from him" and given to these institutions supported by charity. Indeed there has been such a mono-tone running through the editorials on this subject that it is refreshing to hear a different key struck, and that, too, by a master hand.

The *Journal* virtually says it is all bosh, about the doctors being robbed of their practice, and it looks very much that way to us. Suppose, for instance, that there were no charity institutions? "The poor we have always with us." The poor are prone to sickness and to injury. Humanity demands that they shall not be permitted to suffer and die neglected. Who would do the practice? On whom would fall the burden of visiting, prescribing for and attending these thousands of pauper patients? For our part, we would be glad to get rid of the share of it which would fall to us; we would say, "take it, and thanky too."

Hamilton says there has been "an overpreachment of charity to medical students" and "much beckoning into the halls of learning." There he struck the prime source from which this great "evil" arises: *too many doctors!* The process of making them has been perfected, (and "patent applied for"), that is, the process of making them rapidly and cheaply. It is a case of over production. The editor of the *Journal* says: "The doctors are eager to set up small dispensaries," etc. They are eager to do anything that offers a shadow of a chance to make a living, and their necessities drive them to devious ways. The remedy? It is plainly indicated: Cut off the supply of doctors turned out each year by the cheap process. If States must legislate on medicine let them legislate about four-fifths of these doctor-factories out of existence. Then, there will be paying practice for what remains; and the charity institutions will find their legitimate work, and will be a blessing alike to the medical profession and the poor.

